# Composition and evolution of the gut microbiota of growing puppies is impacted by their birth weight

**DOI:** 10.1038/s41598-023-41422-9

**Published:** 2023-09-07

**Authors:** Quentin Garrigues, Emmanuelle Apper, Ana Rodiles, Nicoletta Rovere, Sylvie Chastant, Hanna Mila

**Affiliations:** 1 NeoCare, Reproduction, ENVT, Université de Toulouse, 23 Chemin des Capelles, BP 87614, 31 076 Toulouse Cedex 3, France; 2 Lallemand SAS, Blagnac, France; 3 Department of Health, Animal Science and Food Safety, VESPA, University of Veterinary, 20134 Milan, Italy

**Keywords:** Bacteria, Bacteriology, Clinical microbiology, Microbial communities, Animal physiology, Biomarkers, Risk factors

## Abstract

Low birth weight puppies present an increased risk of neonatal mortality, morbidity, and some long-term health issues. Yet it has not been investigated if those alterations could be linked to the gut microbiota composition and evolution. 57 puppies were weighed at birth and rectal swabs were performed at 5 time points from birth to 28 days of age. Puppies were grouped into three groups based on their birth weight: low birth weight (LBW), normal birth weight (NBW) and high birth weight (HBW). 16S rRNA gene sequencing was used to highlight differences in the fecal microbiota. During the first three weeks, the relative abundance of facultative anaerobic bacteria such as *E. coli, C. perfringens* and *Tyzzerella* was higher in LBW feces, but they catch back with the other groups afterwards. HBW puppies showed higher abundances of *Faecalibacterium* and *Bacteroides* during the neonatal period, suggesting an earlier maturation of their microbiota. The results of this study suggest that birth weight impact the initial establishment of the gut microbiota in puppies. Innovative strategies would be desired to deal with altered gut microbiota in low birth weight puppies aiming to improve their survival and long term health.

## Introduction

In the canine species, despite progress in veterinary medicine, neonatal mortality remains relatively high with around one out of ten puppies dying during the first three weeks of life^1,2^. One major mortality risk factor identified recently in puppies and studied in many other mammalian species is the birth weight of newborns. Specifically, low birth weight (LBW) puppies have an 8 times higher mortality risk attributed to lower energy reserves, thermoregulation and vitality compared to normal-birth weight (NBW) and high-birth weight (HBW) individuals^3,4^. Moreover, this lack of vitality, as evidenced by lower APGAR scores, makes the access to mammary glands for colostrum intake difficult for them, resulting in reduced passive immune transfer, and thus higher risk of infectious diseases^5^. High mortality in LBW represents important ethical and economic challenges for breeders and veterinarians, making the study of early risk factors a priority to improve the wellbeing of these vulnerable newborns. Besides, deficit of passive immune transfer and metabolic issues, gut microbial composition may impact newborn health.

The gastrointestinal tract (GIT) of mammals harbors trillions of bacteria forming the gut microbiota and playing many different roles in the health of their host, promoting the maturation of the immune system, mucosal integrity, and metabolite production^6,7^. Many studies suggest that the early development of the gut microbiota may impact the infant’s growth during the first months of life and his health until adulthood^8^. Thus, altered gut microbiota development can lead to a higher risk of neonatal mortality and diseases, like enterocolitis, and also long-term adverse effects such as the promotion of type-1 diabetes and increased adiposity^9–11^. In humans and piglets, numerous studies have demonstrated that gut microbiota of low birth weight newborns differed from the one with normal birth weight^12,13^, and that manipulating the gut microbiota could improve outcomes like oxidative stress biomarkers or lipid metabolism^14,15^.

Despite the importance of the gut microbiota development in general health and even survival of the individual, the relationship between the birth weight and the gut microbiota development during the first weeks of life have never been studied in the canine species.

The aim of this study was thus to determine potential differences in the gut microbiota richness and composition depending on birth weight in puppies from birth up to 28 days of age. Our hypothesis was that the fecal microbiota of low birth weight puppies differs in diversity and in composition.

## Results

### Description of the population studied

Among the 57 puppies studied, 14 were LBW, 29 NBW and 14 HBW. Twenty-three puppies were Australien Shepherd, 17 were Golden Retriever, 12 were Labrador Retriever and 5 were White Swiss Shepherd. Birth weights ranged from 173 to 523 g with a mean value of 399 g ± 82 g (Table 1). Sex ratio (male/female) was of 1.11, with 30 male and 27 female puppies included in the study. The APGAR score, available for 44 puppies (77%), ranged from 6 to 10 (mean = 8.4 ± 1), with significant differences in the APGAR score observed among birth weight quartiles. At birth, LBW puppies showed a significantly lower APGAR score (n = 11, mean = 7.6 ± 0.9) than NBW and HBW puppies (respectively, n = 23, mean = 8.7 ± 1 and n = 10, *p* = 0.008 and mean = 8.8 ± 0.8; *p* = 0.001).

**Table 1:** Characteristics of puppies included in the study.

Birth weight quartiles	Number of puppies	Birth weight (g) (mean ± SD)	Minimum birth weight (g)	Maximum birth weight (g)	Sex ratio (M/F)	APGAR score (mean ± SD)
Q1	14	289 ± 52	173	375	1.33	7.6 ± 0.9 (n = 11)
Q2_3	29	390 ± 38	333	495	0.93	8.7 ± 1.0 (n = 15)
Q4	14	462 ± 32	426	523	1.33	8.8 ± 0.8 (n = 8)
Total population	57	383 ± 73	173	523	1.11	8.4 ± 1.0 (n = 10)

### Evolution of the fecal microbiota composition of the puppies over the first 28 days of life

**Figure 1:**
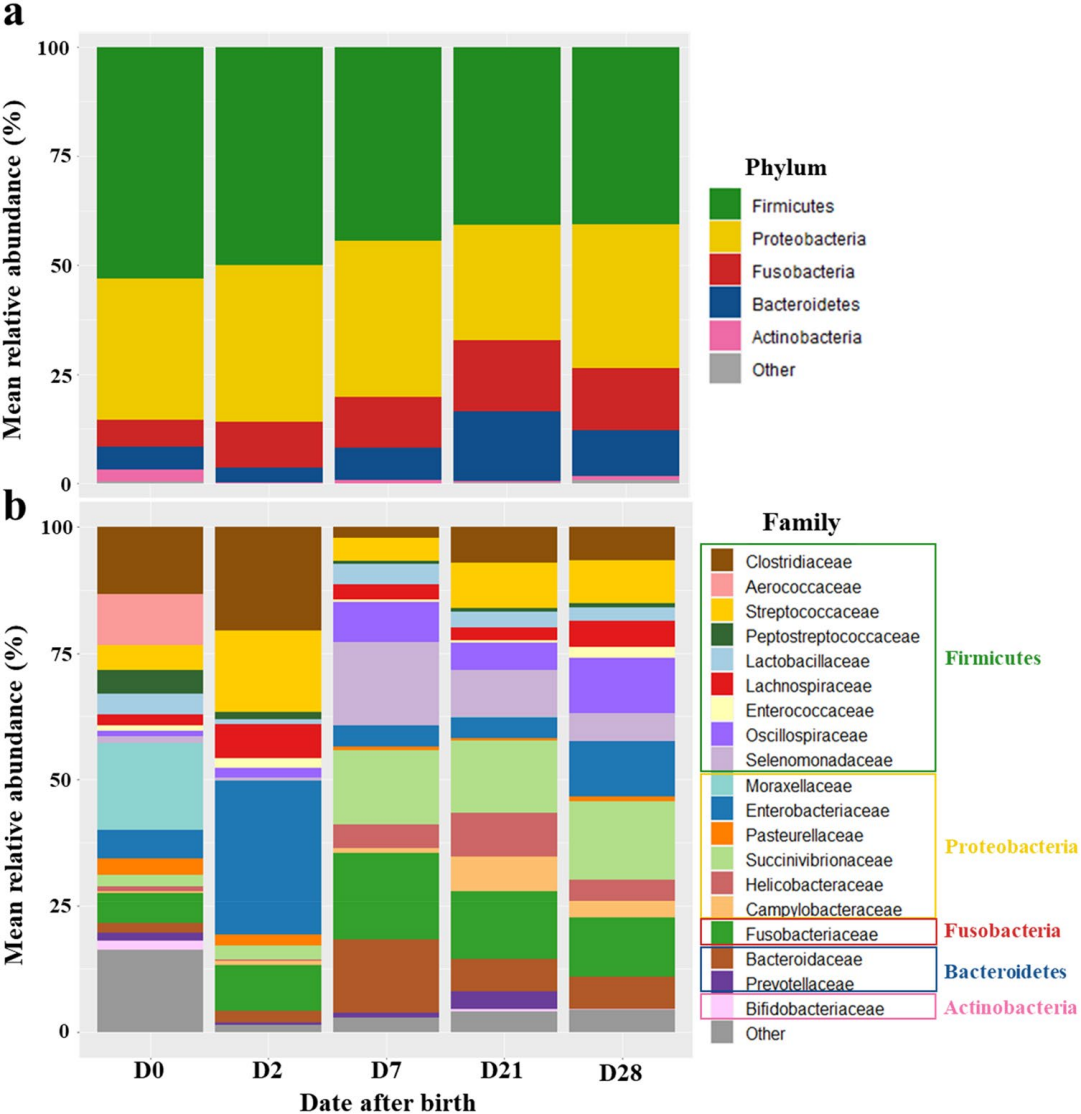
Evolution of the mean relative abundance of bacteria depending on the age of the 57 studied puppies at (**a**) the phylum level and (**b**) the family level. The 18 most abundant families at all dates, plus *Bifidobacteriaceae*, are highlighted. All the remaining families are summed in the “Other” category.

A total of 7,806,736 sequences were used for the analysis. After cleaning, filtration and affiliations of the sequencing reads, 268 ASVs were identified, composed of 14 phyla, 22 classes, 42 orders, 85 families, 140 genera. At all dates during the first 28 days of life, Firmicutes, Proteobacteria, Fusobacteria, Bacteroidetes and Actinobacteria were the five main phyla representing the puppies’ microbiota. Firmicutes and Proteobacteria dominated the fecal microbiota at all dates, with an abundance ranging respectively from 41% (D28) to 53% (D0) (mean at all time points of 45.8% ± 17.4) and from 26% (D21) to 36% (D2) (mean at all times of 32.6% ± 18.3) of the total relative abundance (Fig. 1). The following phyla with the highest abundance were Fusobacteria (mean of 11.8% ± 10.9), Bacteroidetes (mean of 8.6% ± 9.5) and Actinobacteria (mean of 1.1% ± 3.0). Proteobacteria and Fusobacteria relative abundances remained quite stable over time (respectively around 33% and 11%). Bacteroidetes was the phylum with the biggest shifts over time, as its relative abundance showed an increase from 5% at birth to 16% at D21 before shifting down at around 10% on D28. Actinobacteria only had a relative abundance of 3% at D0 before being almost absent from D2 to D28. The most prominent families at all dates of the Firmicutes phylum were *Clostridiaceae*, *Streptococcaceae*, *Lactobacillaceae*, *Oscillospiraceae*, *Lachnospiraceae* and *Selenomonadaceae*. Proteobacteria was mostly represented by *Enterobacteriaceae*, *Succinivibrionaceae*, *Helicobacteraceae* and *Campylobacteraceae*. Fusobacteria and Actinobacteria were mostly represented by one family, respectively *Fusobacteriaceae* and *Bifidobacteriaceae*. Finally, the two mains families of the Bacteoidetes phylum were *Bacteroidaceae* and *Prevotellaceae* (Fig. 1).

A linear mixed effects model of the phylum abundance with time as fixed effect and puppy ID as the random factor was performed, and significant differences in abundance across time for all six phyla were found with *p*-value < 0.005.

### Variation of alpha diversity and beta diversity over the study

**Figure 2:**
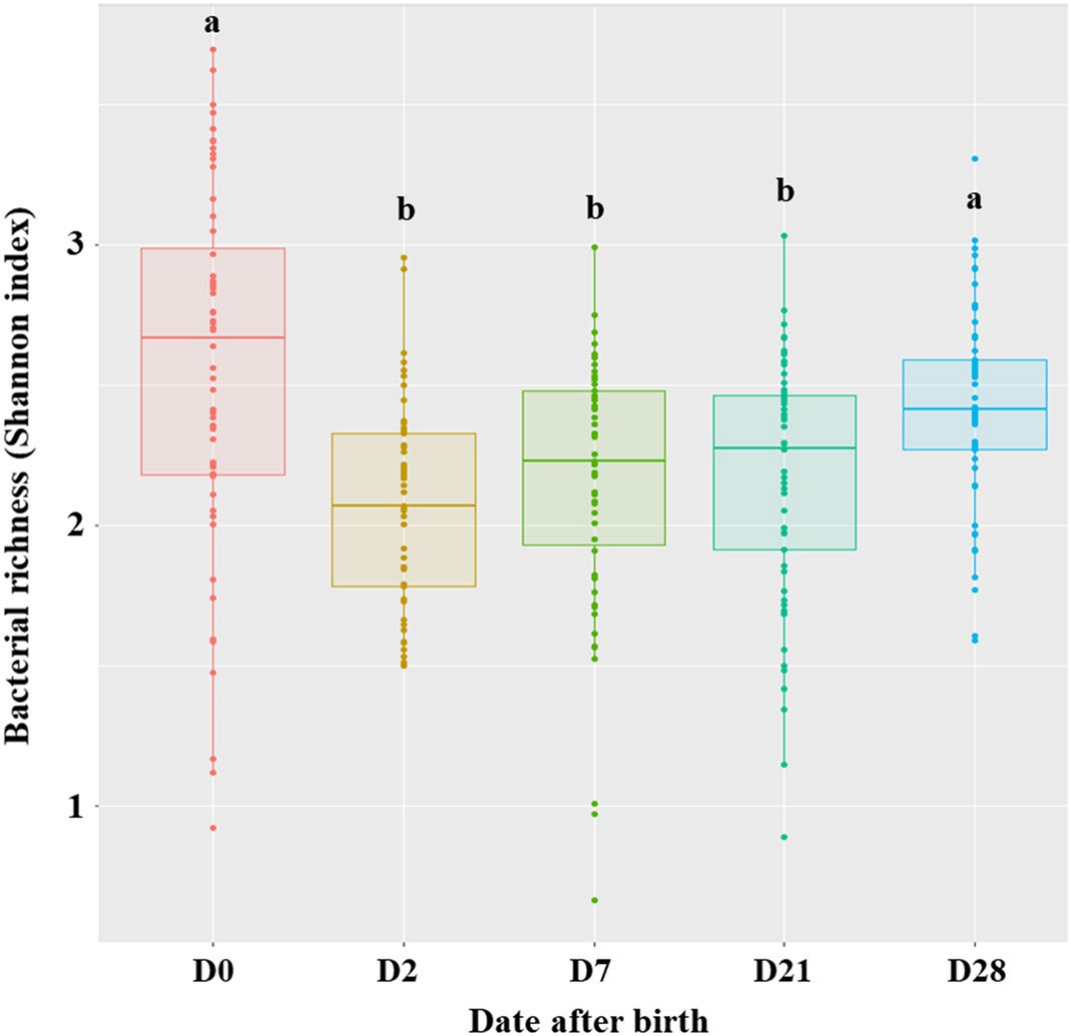
Bacterial richness of fecal microbiota according to the age of puppies. Significant differences observed in Shannon indices are identified with Tukey HSD letters. Boxes with different letters are significantly different (*p* < 0.05).

**Figure 3:**
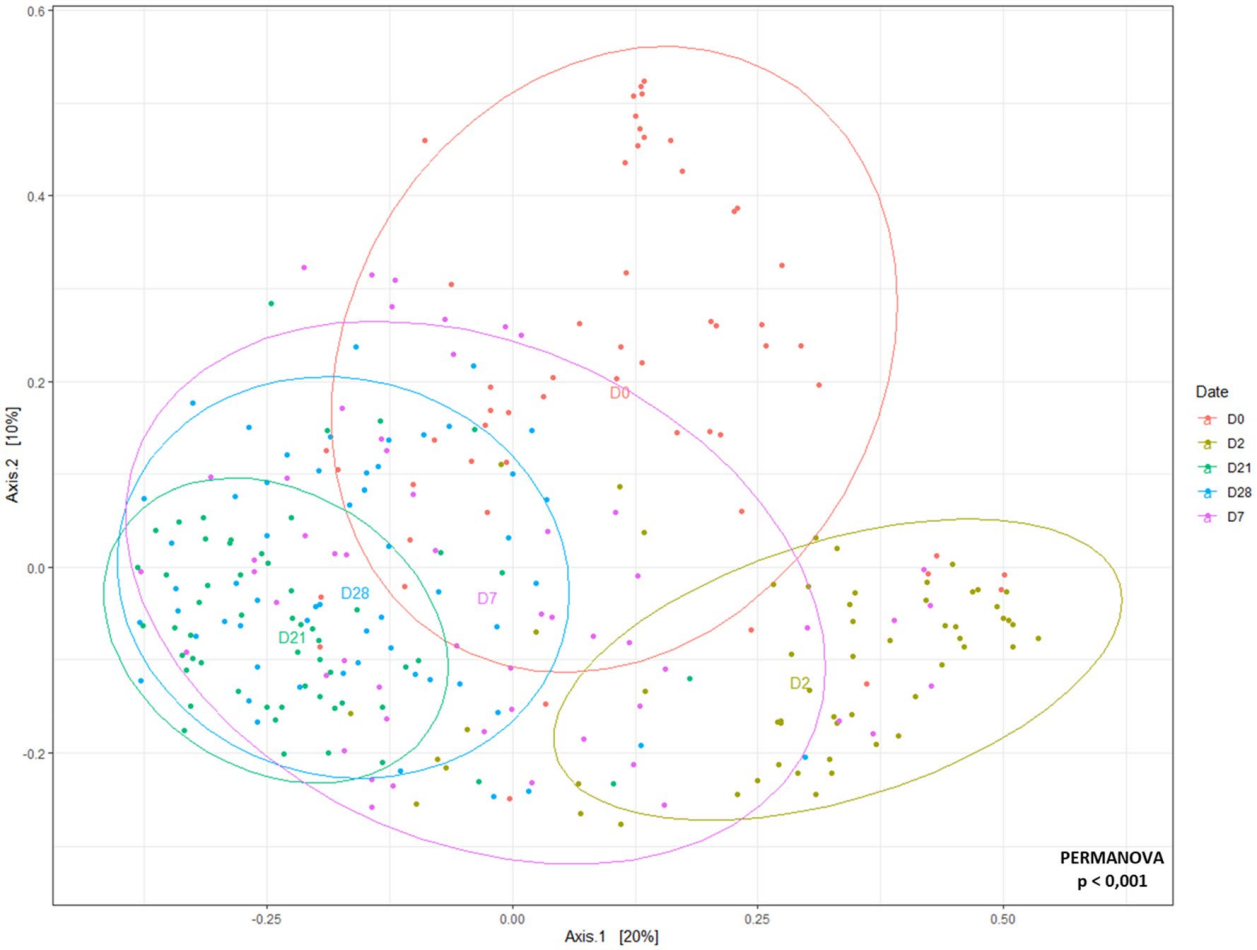
Evolution of fecal microbiota composition with puppies’ age. MDS plot shows the evolution and dissimilarities of puppies’ fecal microbiota composition with age, calculated using Bray–Curtis distances. Each point represents an individual puppy sample, positioned on the plot based on the similarity of its microbiota communities with other samples. Ellipses are based on 95% confidence intervals and standard error. Similarity of bacterial communities is evaluated based on taxa similarity and abundance. (*p* < 0.001).

The age of the puppies significantly affected the number of observed ASVs, Shannon and InvSimpson diversity indices (*p* < 0.001). As results were identical, independently of the indices used, only Shannon was kept for graphical visualization. The bacterial richness and evenness significantly decreased from D0 to D2 (*p* < 0.001), remained stable between D2 and D21 before increasing from D21 up to D28, but still remaining lower than D0 (Fig. 2). The average number of observed ASVs went from 57 ± 20.8 at D0 to 29 ± 9.3 at D21 before increasing again up to 39 ± 100 at D28, with the same trend for Shannon index. The age of the puppies also had a significant effect in the evolution of the community composition (*p* < 0.001) based on the PERMANOVA results using Bray–Curtis distances (Fig. 3). The microbial communities could be separated into two main clusters. The first cluster included all D2 samples, while the second cluster was comprised of samples from D21 and D28. Interestingly, D0 and D7 showed a higher variation, with samples present in both clusters. The size of the ellipses also allowed to highlight a higher variation in the composition of the microbiota among puppies at D7 when compared to other dates where ellipses were smaller.

### Associations between birth weight quartiles and bacterial richness and diversity

**Figure 4:**
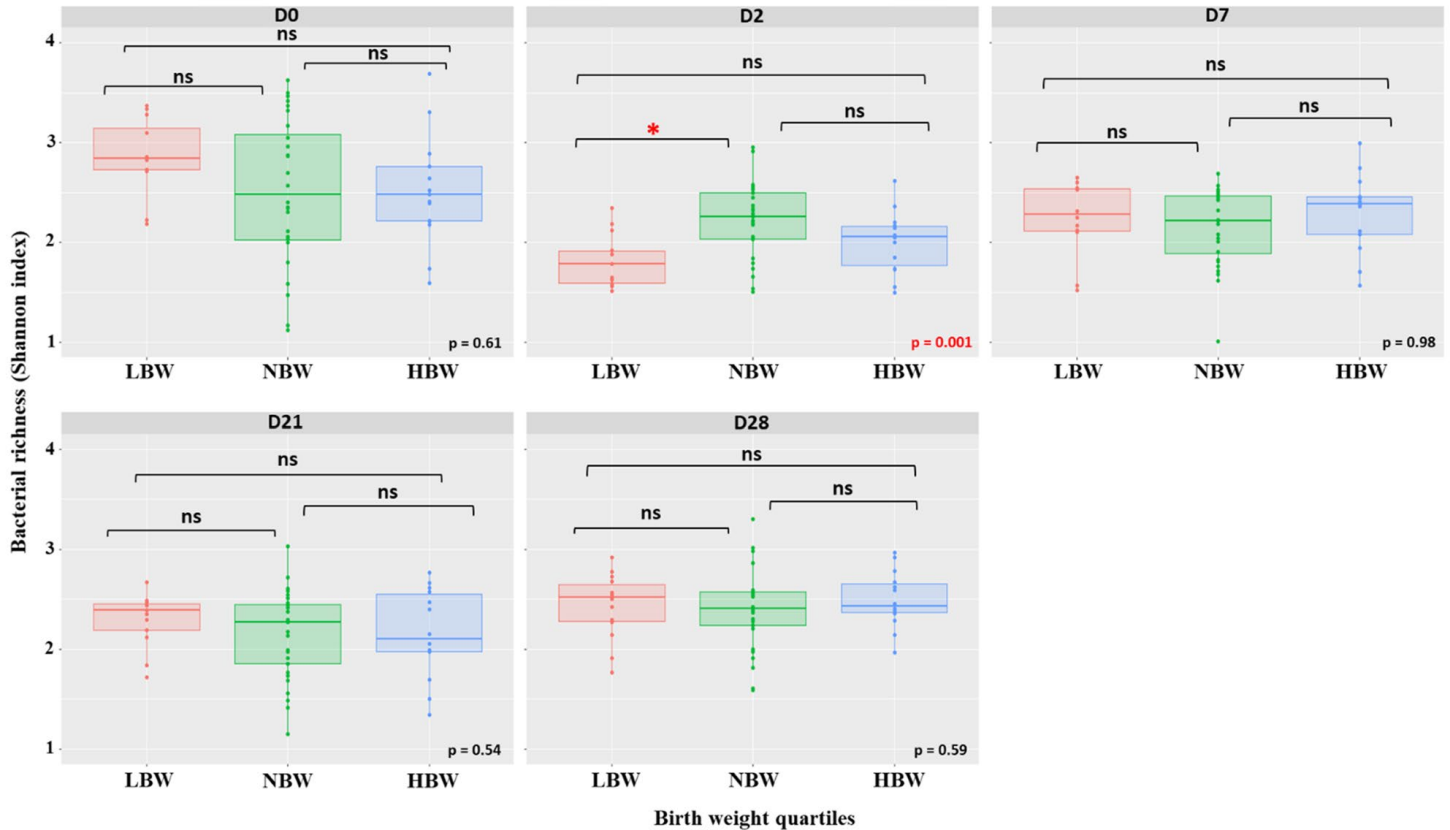
Bacterial richness (Shannon diversity index) of fecal microbiota according to birth weight quartile over the first 2 months of life (D0: birth). ns: non significant.

Among different time points from D0 up to D28, D2 was the only date when a significant difference in bacterial richness (Shannon index) was observed between birth weight quartile groups (*p* = 0.001) (Fig. 4). Tukey’s post-hoc test indicated that LBW puppies had a significantly lower bacterial richness than NBW (*p* < 0.001), but not HBW (*p* = 0.29).

**Figure 5:**
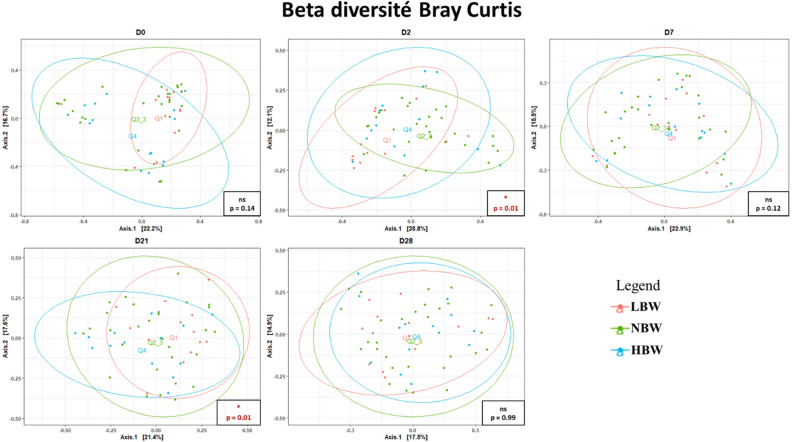
Beta diversity of fecal microbiota (Bray–Curtis index) depending on birth weight. MDS plots showing the evolution and dissimilarities of puppies’ fecal microbiota composition based on their birth weight quartile groups calculated using Bray–Curtis distances. Each point represents an individual puppy sample, positioned on the plot based on the similarity of its microbiota community with other samples. The closer points are on the plot, the more related their bacterial communities are, based on taxa similarity and abundance. ns, Non significant.

At D2 but also at D21, there was a significant difference in beta diversity (Bray–Curtis index) in the overall bacterial composition depending on quartile groups (respectively *p* = 0.006, R^2^ = 0.08 and *p* = 0.012, R^2^ = 0.07; Fig. 5). According to post hoc tests, LBW puppies presented different bacterial communities compared with NBW at D2 (*p* adjusted = 0.03), and between LBW and HBW at D21 (*p* adjusted = 0.04). No differences in the microbial community structure were noticed between quartile groups at D0, D7 or D28.

### Differences in the fecal microbiota composition according to birth weight of puppies over the first month of life

Significant differences in taxa relative abundance between birth weight quartiles were observed and classed by age in Table 2. D2 and D21 are the only dates when more than three taxa showed significant differences in relative abundance and high VIP score. A further day by day analysis was performed to better understand the differences between quartiles for taxa with the highest biological interests, VIP and relative abundance. *p*-values of the fixed covariate effects and Tukey’s post-hoc tests are available in supplementary Table S1.

**Table 2:** List of taxa with significant differences of relative abundance between birth weight quartiles and with a VIP score higher than 1.2 (with the exception of 4 taxa having a lower VIP score).

Age	Bacterial taxa	Mean relative abundance in Q1 (± SD)	Mean relative abundance in Q2_3 (± SD)	Mean relative abundance in Q4 (± SD)	*p*-val quartile	VIP score
D0	*Pasteurellaceae*	5.22 (± 8.77)	3.99 (± 9.05)	2.45 (± 4.62)	0.025	1.45
	*Delftia*	0.56 (± 1.60)	0.03 (± 0.16)	0.04 (± 0.07)	0.030	3.24
D2	*Porphyromonadaceae*	0.00 (± 0.00)	0.14 (± 0.41)	< 0.01 (± 0.03)	0.045	1.34
	*Clostridiaceae*	23.09 (± 11.67)	17.53 (± 9.91)	17.00 (± 12.60)	0.020	0.94
	*Clostridium*	22.02 (± 11.71)	16.46 (± 9.68)	16.31 (± 12.34)	0.023	0.96
	*Roseburia*	0.00 (± 0.00)	0.04 (± 0.16)	0.15 (± 0.40)	0.006	1.97
	*Tyzzerella*	7.10 (± 10.17)	1.48 (± 3.41)	2.15 (± 2.56)	0.015	2.06
	*Faecalibacterium*	< 0.01 (± 0.03)	0.03 (± 0.14)	0.03 (± 0.05)	0.000	1.85
	*Phascolarctobacterium*	0.26 (± 0.60)	0.48 (± 0.95)	0.05 (± 0.11)	0.013	0.67
	*Selenomonadaceae*	0.94 (± 1.86)	0.48 (± 0.69)	0.17 (± 0.37)	0.022	1.86
	*Megamonas*	0.94 (± 1.86)	0.47 (± 0.69)	0.17 (± 0.37)	0.012	1.90
	*Fusobacteriaceae*	4.68 (± 10.00)	14.15 (± 11.56)	8.26 (± 12.45)	0.007	1.92
	Unknown genus of *Fusobacteriaceae*	2.65 (± 7.36)	5.45 (± 6.19)	2.84 (± 6.15)	0.002	2.39
	*Sutterellaceae*	0.02 (± 0.03)	0.21 (± 0.43)	0.08 (± 0.25)	0.001	1.76
	*Sutterella*	0.02 (± 0.03)	0.21 (± 0.43)	0.08 (± 0.25)	0.001	1.75
	*Enterobacteriaceae*	38.45 (± 12.26)	25.11 (± 12.38)	27.59 (± 11.94)	0.002	1.90
	*Escherichia*	36.80 (± 11.96)	24.76 (± 12.29)	26.43 (± 11.77)	0.004	1.83
D7	*Enterobacteriaceae*	12.11 (± 19.22)	9.62 (± 12.75)	11.98 (± 12.88)	0.049	#N/A
D21	*Enterobacteriaceae*	7.45 (± 6.35)	2.98 (± 4.16)	3.05 (± 1.52)	0.000	2.32
	*Escherichia*	7.11 (± 5.87)	2.97 (± 4.16)	3.02 (± 1.50)	0.000	2.21
	*Lachnospiraceae*	5.13 (± 6.31)	1.50 (± 3.32)	1.92 (± 3.61)	0.026	2.12
	*Phascolarctobacterium*	1.44 (± 1.87)	2.45 (± 2.68)	2.12 (± 2.95)	0.028	1.37
	*Acidaminococcaceae*	1.44 (± 1.87)	2.45 (± 2.68)	2.12 (± 2.95)	0.029	1.23
	*Bacteroidaceae*	14.36 (± 8.16)	11.34 (± 8.19)	22.21 (± 8.74)	0.046	1.68
	*Bacteroides*	14.36 (± 8.16)	11.34 (± 8.19)	22.21 (± 8.74)	0.049	1.81
D28	*Deferribacteraceae*	0.72 (± 1.83)	0.08 (± 0.18)	2.18 (± 4.00)	0.009	3.05
	*Mucispirillum*	0.72 (± 1.83)	0.08 (± 0.18)	2.18 (± 4.00)	0.011	2.95
	*Veillonellaceae*	1.99 (± 2.94)	0.99 (± 2.08)	1.46 (± 2.19)	0.044	1.40

“*p*-val Quartile” corresponds to the *p*-value of the birth weight quartile fixed effect. A value of “N/A” means the variation of the taxa between birth weight quartiles was too low for the VIP score to be calculated.

**Figure 6:**
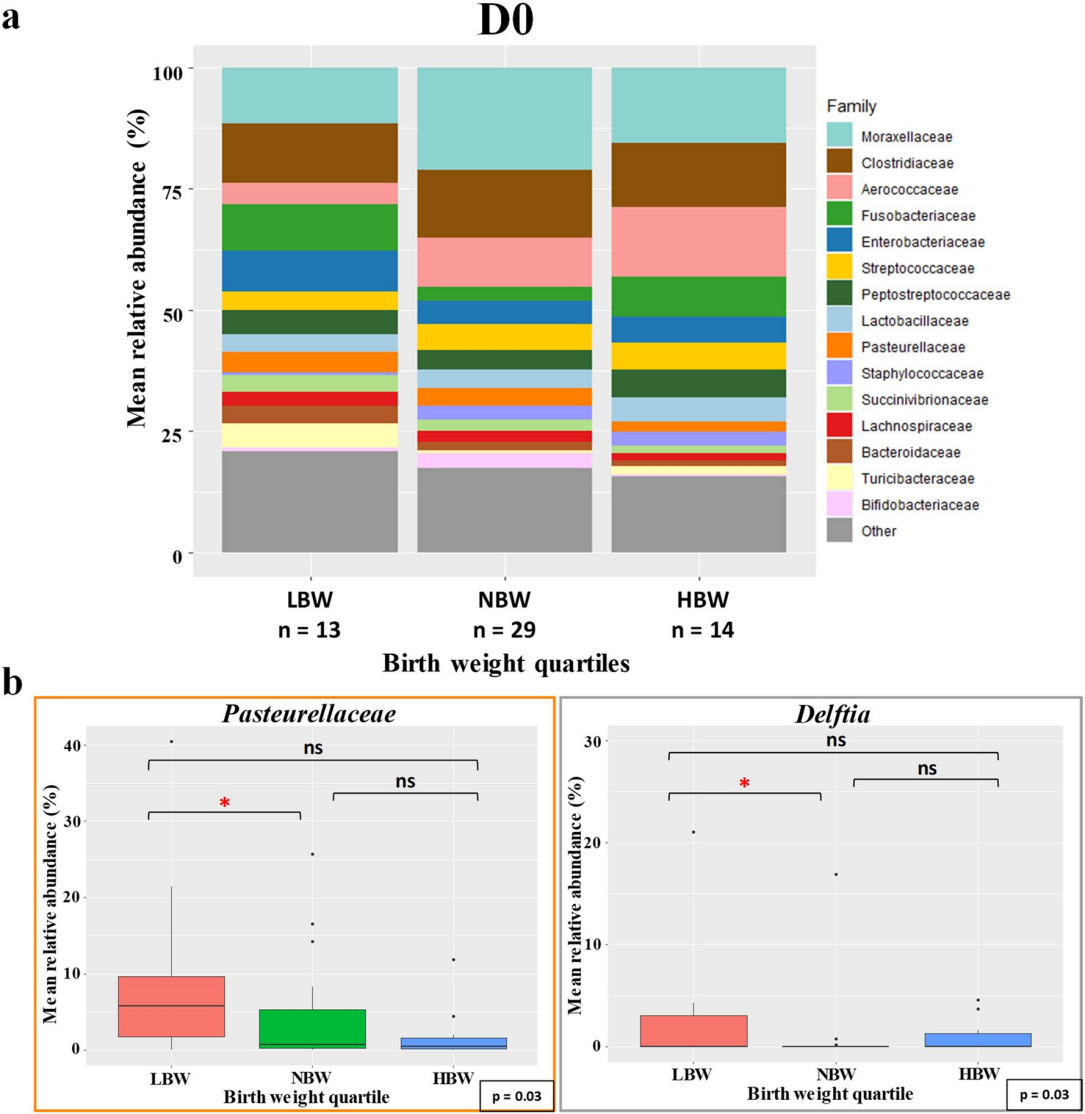
Taxa relative abundance at day 0 (n = 56 puppies) (**a**) Stacked bar plot of the mean relative abundances of the 15 most abundant classified families for each quartile on the day of birth. The 16th family “Other” is the sum of the relative abundance of all remaining families. (**b**) The family *Pasteurellaceae* and the genus *Delftia* were selected for a focused box plot analysis. A red asterisk (*) indicates a significantly different relative abundance (adjusted *p* ≤ 0.05) between two quartile groups after Tukey’s post-hoc test, while an orange dot (**·)** indicates a trend (adjusted 0.1 ≥ *p* > 0.05).

At D0, 2 families of strict aerobic bacteria, *Moraxellaceae* and *Aerococcaceae* represented 24% of the total relative abundance of the gut microbiota of puppies (Fig. 6). No significant differences in abundance of those two families between birth weight quartiles have been observed, but a trend with LBW puppies having lower abundances compared to other groups. On the opposite, some strict and facultative anaerobes bacteria had higher relative abundances in LBW, such as *Pasteurellaceae* (*p* = 0.03).

**Figure 7:**
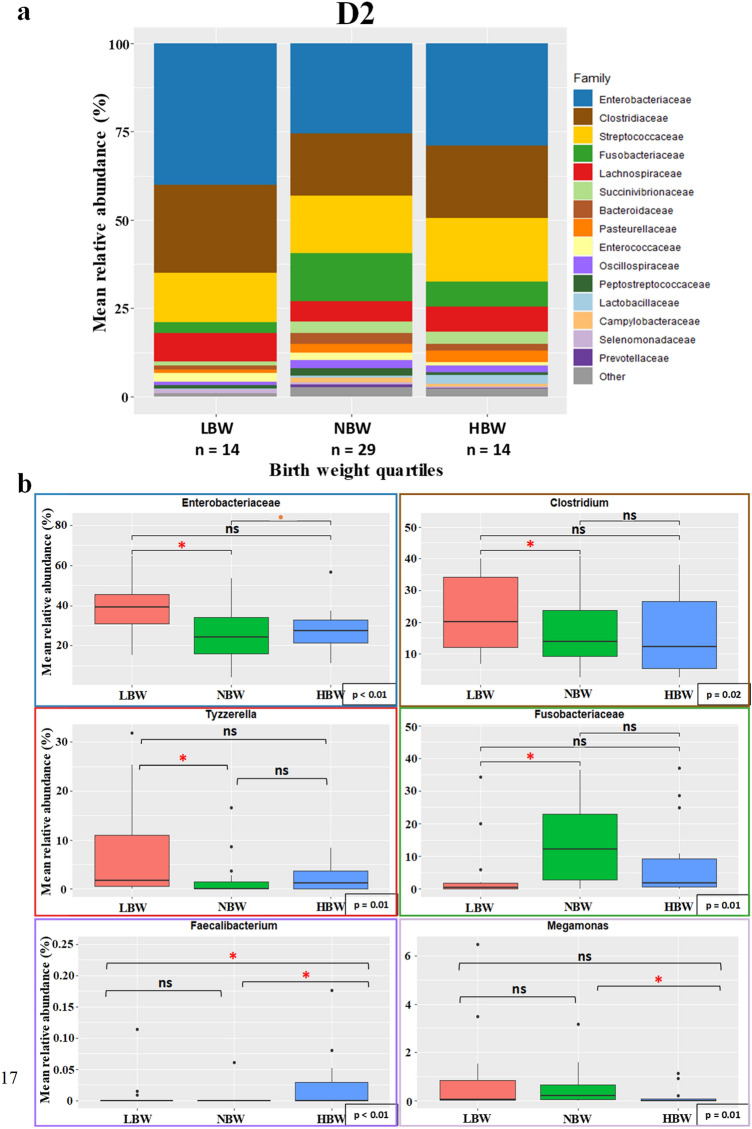
Taxa relative abundance at day 2 (n = 57 puppies) (**a**) Stacked bar plot of the mean relative abundances of the 15 most abundant classified families for each quartile at the second day after birth. The 16th family “Other” is the sum of the relative abundance of all remaining families. (**b**) The families *Enterobacteriaceae* and *Fusobacteriaceae* and the genera *Clostridium*, *Tyzzerella*, *Faecalibacterium* and *Megamonas* were selected for a focused box plot analysis. A red asterisk (*) indicates a significantly different relative abundance (adjusted *p* ≤ 0.05) between two quartile groups after Tukey’s post-hoc test, while an orange dot (·) indicates a trend (adjusted 0.1 ≥ *p* > 0.05).

As observed in Table 2, the age when puppies had the most observed differences in microbial composition depending on birth weight quartiles was D2 (Fig. 7). LBW puppies showed a significantly higher relative abundance of *Enterobacteriaceae* (mostly comprised of *E. coli*), *Clostridiaceae* (mostly comprised of the *Clostridium* genus), and *Tyzzerella*, a genus from the *Lachnospiraceae* family (respectively *p* = 0.002, *p* = 0.023 and *p* = 0.015) (Fig. 7). They also showed a significantly lower relative abundance of *Phascolarctobacterium* compared to NBW (*p* = 0.013). Both *Clostridiaceae* and *Phascolarctobacterium* had a VIP score below 1.2, but were kept in the analysis due to their biological interest. The HBW puppies had a significantly higher relative abundance of *Faecalibacterium* compared to the other two groups (*p* < 0.001) and a significantly lower relative abundance of *Megamonas* (*p* = 0.012). Finally, NBW puppies had a significantly higher relative abundance of *Fusobacteriaceae* (*p* = 0.007).

**Figure 8:**
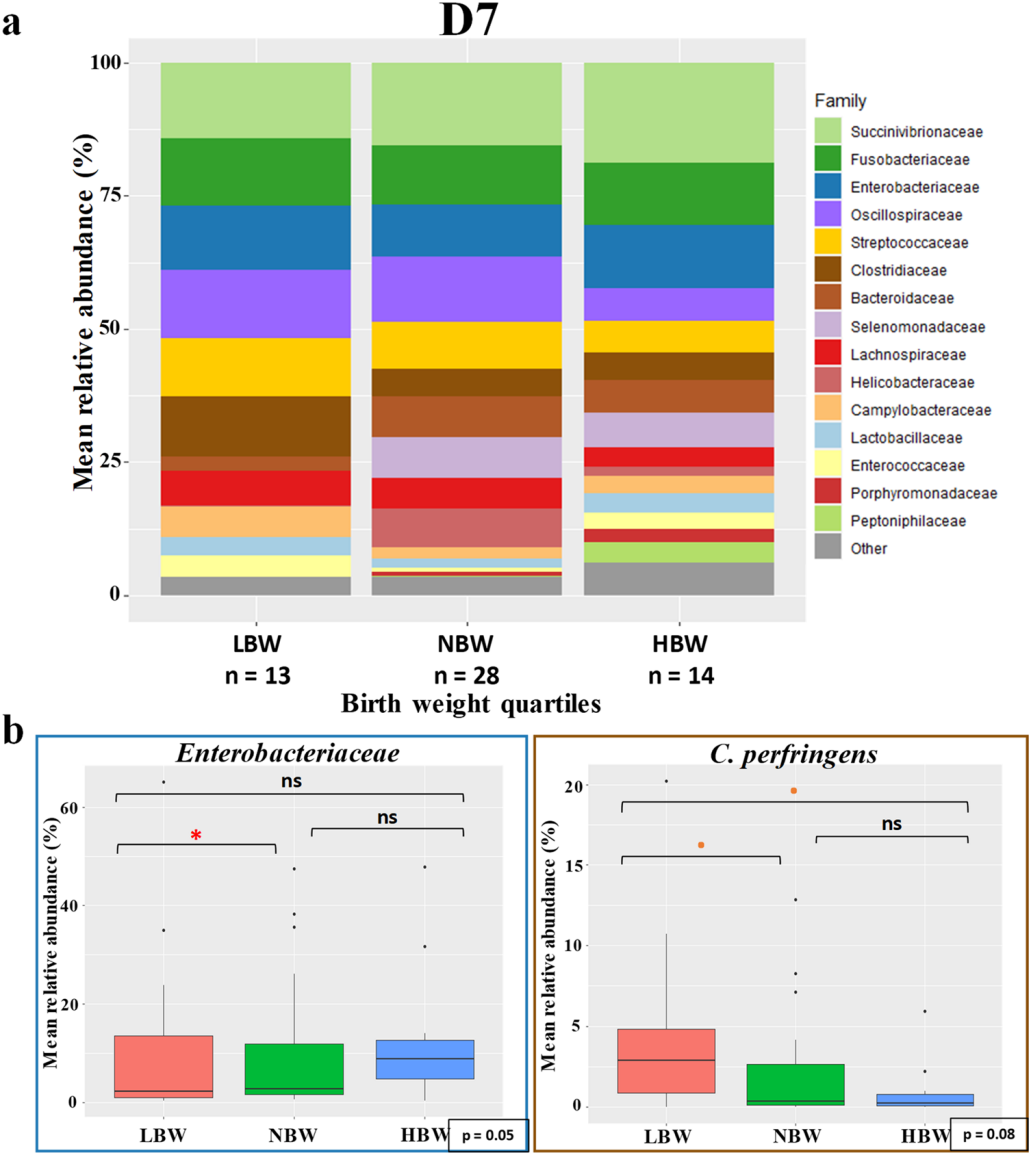
Taxa relative abundance at day 7 (n = 55 puppies) (**a**) Stacked bar plot of the mean relative abundances of the 15 most abundant classified families for each quartile at the seventh day after birth. The 16th family “Other” is the sum of the relative abundance of all remaining families. (**b**) The *Enterobacteriaceae* family and the species *Clostridum perfringens* were selected for a focused box plot analysis. A red asterisk (*) indicates a significantly different relative abundance (adjusted *p* ≤ 0.05) between two quartile groups after Tukey’s post-hoc test, while an orange dot (**·)** indicates a trend (adjusted 0.1 ≥ *p* > 0.05).

At D7, the linear mixed model highlighted a significantly higher relative abundance of *Enterobacteriaceae* (mainly *E. coli*) in LBW puppies compared to NBW (*p* = 0.043), but the sum VIP of this taxa was below 1.2 (Fig. 8). The *Clostridiaceae* family also showed a higher abundance in LBW, but with no statistical difference. However, there was a trend with *C. perfringens* having higher relative abundance in LBW (*p* = 0.077).

**Figure 9:**
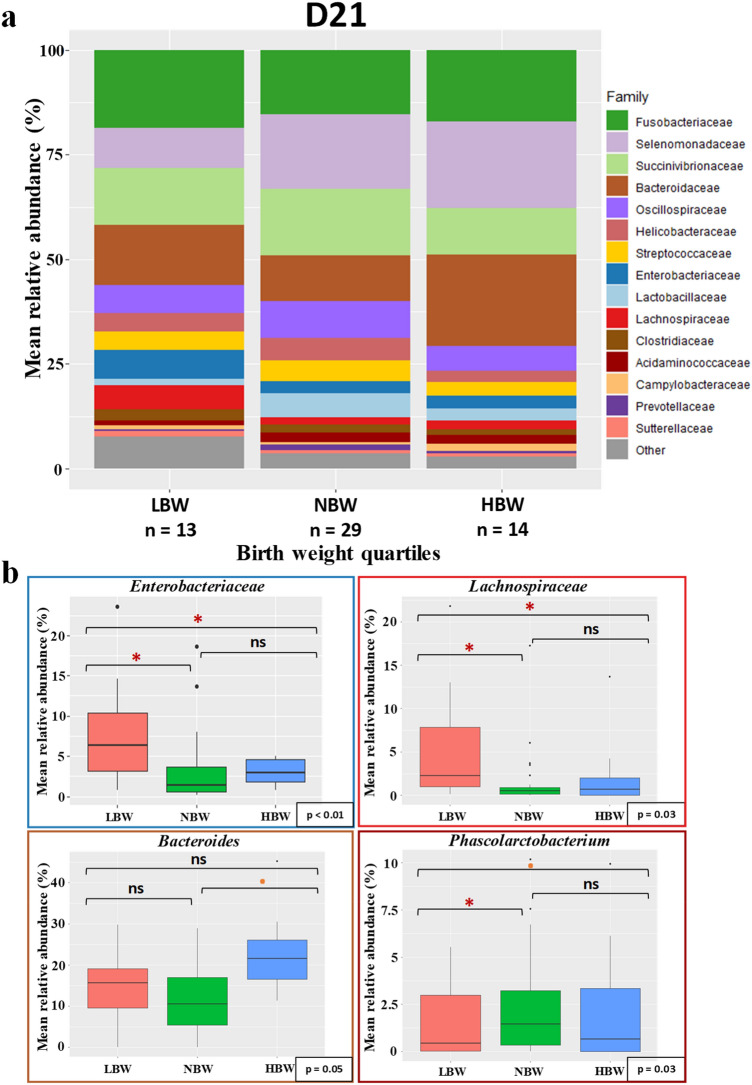
Taxa relative abundance at day 21 (n = 56 puppies) (**a**) Stacked bar plot of the mean relative abundances of the 15 most abundant classified families for each quartile at the twenty-first day after birth. The 16th family “Other” is the sum of the relative abundance of all remaining families. (**b**) The *Enterobacteriaceae* and *Lachnospiraceae* families and the *Bacteroides* and *Phascolarctobacterium* genera were selected for a focused box plot analysis. A red asterisk (*) indicates a significantly different relative abundance (adjusted *p* ≤ 0.05) between two quartile groups after Tukey’s post-hoc test, while an orange dot (**·**) indicates a trend (adjusted 0.1 ≥ *p* > 0.05).

At D21, LBW puppies showed a significantly higher abundance of *Enterobacteriaceae* (mostly comprised of *E. coli*) and *Lachnospiraceae* with a significant difference compared to both NBW (respectively *p* < 0.001 and *p* = 0.038) and HBW (respectively *p* = 0.035 and *p* = 0.033) (Fig. 9). Once again, they also showed a lower relative abundance of *Phascolarctobacterium* compared to NBW (*p* = 0.024). HBW in the other hand had a significantly higher relative abundance of *Bacteroidaceae*, of which mostly *Bacteroides* (*p* = 0.049).

**Figure 10:**
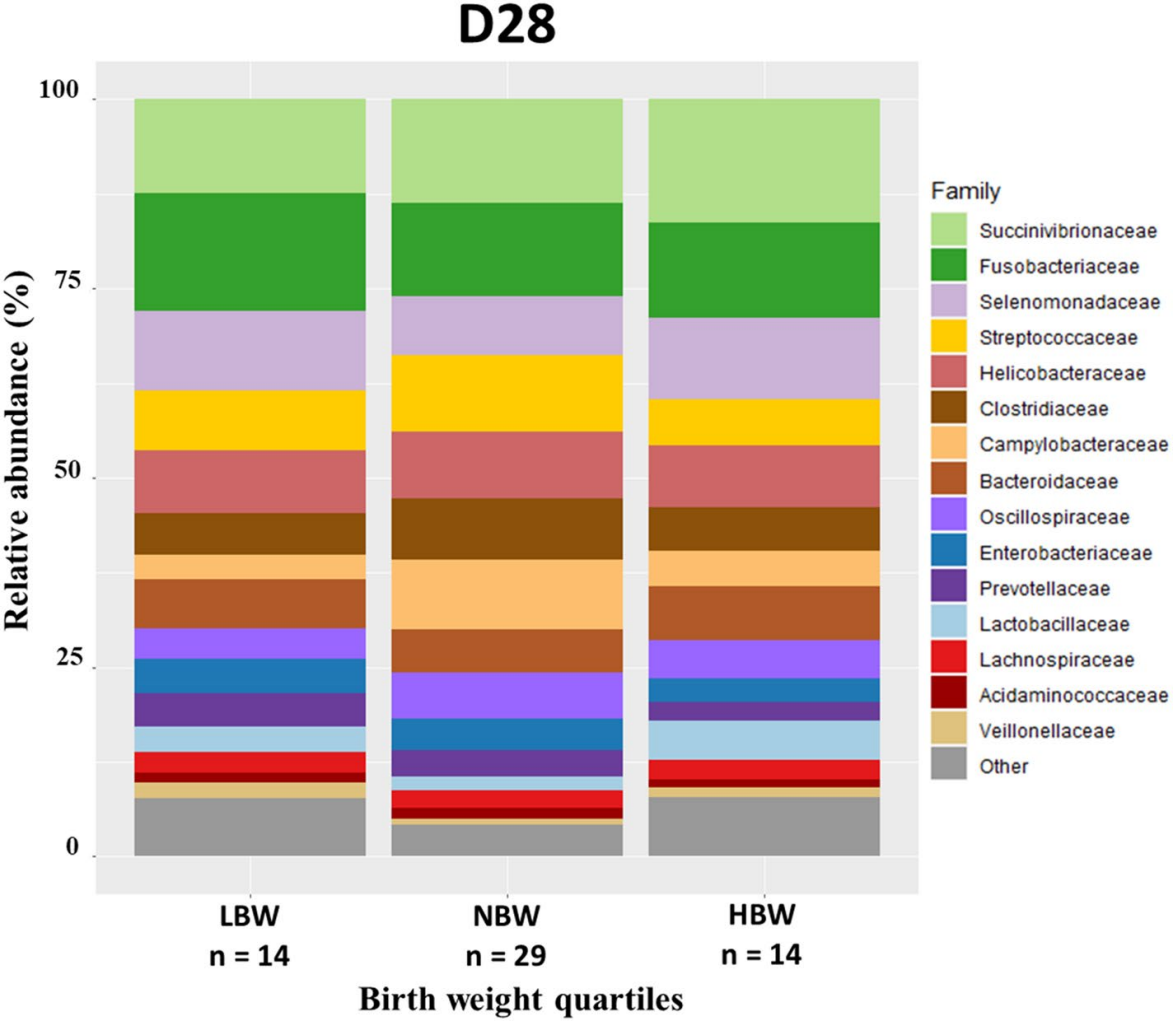
Taxa relative abundance at day 28 (n = 57 puppies) Stacked bar plot of the mean relative abundances of the 15 most abundant classified families for each quartile during the second month of life. The 16th family “Other” is the sum of the relative abundance of all remaining families.

At D28, , only a few differences were noted in the microbiota composition between the three groups according to puppies’ birthweight (Fig. 10). Those differences concerned the HBW puppies versus other groups, of which a higher relative abundance of *Mucispirillum* and *Veillonellaceae*observed (*p* = 0.011 and *p* = 0.04).

Stacked bar plots per individual for each time points were created to observe the inter-individual variability between puppies. These plots are available in Supplementary file S2.

## Discussion

The present study evidenced that gut microbiota varies according to birth weight of puppies. These results suggest that, not only metabolic disturbances, but also intestinal dysbiosis may contribute to the higher neonatal morbidity and mortality of low-birth weight (LBW) puppies. To the authors’ knowledge, this is the first study highlighting such findings.

Firstly, significant changes in the gut microbial communities with age were observed in our study. Bacterial richness significantly decreased between D0 and D2 then increased from D2 to D28, with the most significant increase occuring between D21 and D28. Moreover, the important shifts in the microbial composition were noticed mostly during the first days after birth. As one of the main factor shaping the gut microbiota composition and diversity, puppies’ age has already been reported in the literature, but samples were either not collected at birth or during the first week of life (D7)^16–18^. Thus, our study added insights on the microbiota development, being the first to give a clear picture of the microbiota development on the first days after birth (D0, D2 and D7). Similar results were observed in humans and piglet where the composition of the microbiota shows important shifts in its communities between the first days and the following weeks^19,20^.

When looking at beta diversity, two main microbial profiles were observed. The first one corresponded to the microbiota profile at a very young age, around the first week of life, when it is highly dynamic and unstable, with the largest inter‐individual composition observed. At that time, environmental conditions are favourable for aerobes and facultative anaerobes (*Moraxellaceae*, *Clostridiaceae*, *Aerococcaceae*, *Enterobacteriaceae*). The second profile corresponded to a more mature and anaerobic gastrointestinal tract (GIT) with samples from D21 and D28, mainly populated by bacteria such as *Bacteroidaceae*, *Selenomonadaceae* and *Succinivibrionaceae*. The dissimilarity between those two clusters could be drafted by nutritional shifts taking place in puppies during the first weeks of life. In our study, puppies were fed milk exclusively until 14 days of life, whereas older puppies had access to solid food (kibbles). Previous studies demonstrated that the transition from milk to a solid diet is usually followed by an increase in bacterial diversity^16,21,22^. Moreover, dogs fed a high carbohydrate diet (kibbles) present a high Bacteroidetes/Firmicutes ratio^23,24^, also observed in our study after D21. An increase of the Bacteroidetes phylum, and particularly *Prevotella* and *Bacteroides* genera, as noted in our population on day 21, is known to produce important short-chain fatty acids from carbohydrates and glycans^25,26^. Short-chain fatty acids play a major role in the maintenance of gut and immune homeostasis, which could also explain the second cluster at D21.

**Figure 11:**
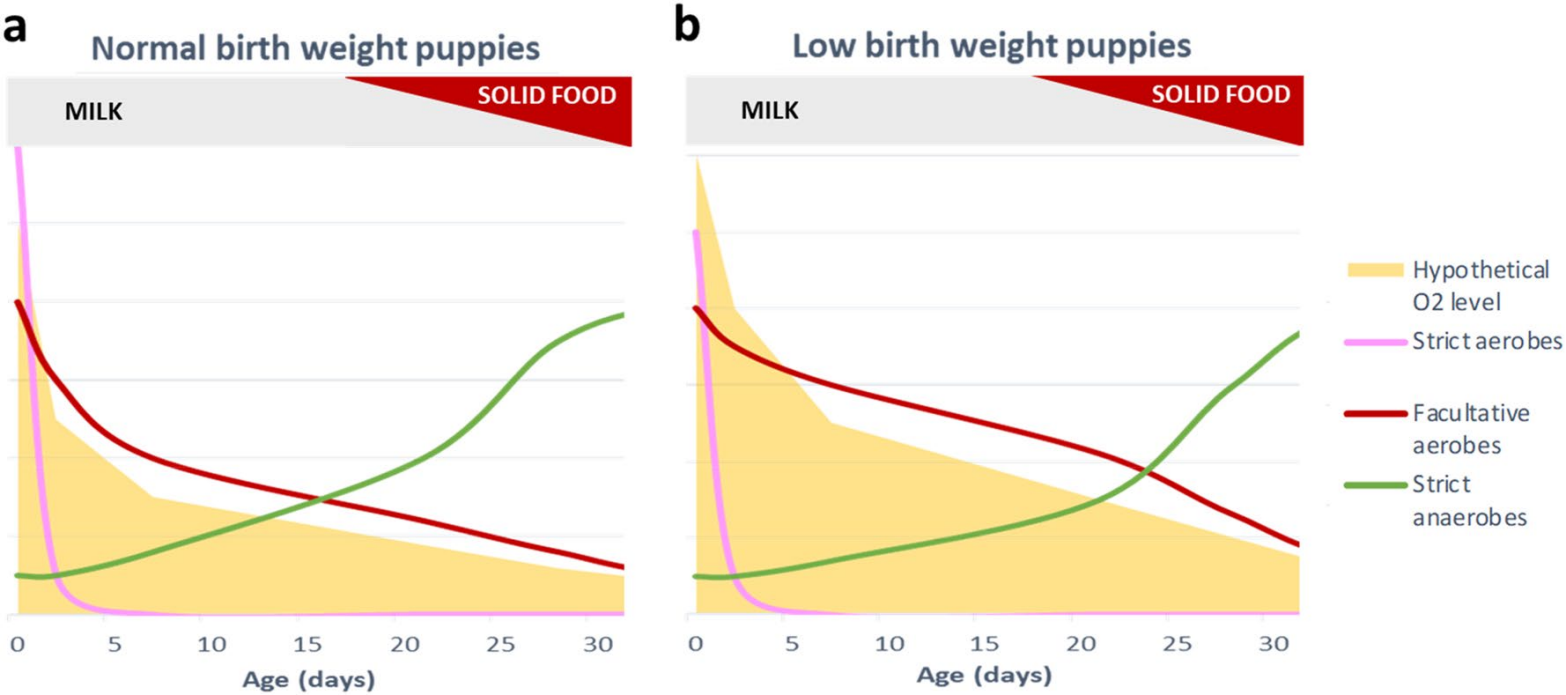
Proposed model based on our results of the evolution of strict aerobes (pink), facultative aerobes (red) and strict anaerobes (green) bacteria types in the GIT of puppies from birth to 1 month old relative to hypothetical O_2_ intestinal level (yellow). The bacterial abundances are representative of the results, while the level of oxygen represents an assumption based on literature. (**a**) In NBW puppies (**b**) In LBW puppies.

In addition to nutritional transition, the two main bacterial profiles could also be explained by physiological shifts occuring in the GIT. Indeed, it has been shown in most mammals that at birth the GIT is filled with oxygen. The maturation of the GIT results in the establishment of an anaerobe environment, thanks to the concomitant action of oxygen-consuming bacteria (i.e., facultative anaerobes) and of colonocyte metabolism, and in particular the beta-oxydation of fatty acids^27^. At the beginning, the positive redox potential in the gut makes the perfect environment for strict and facultative aerobic bacteria (mainly belonging to Proteobacteria and Firmicutes phyla) to settle^28–30^. As those bacteria and the colonocytes consume oxygen, strict aerobic bacteria are quickly replaced with opportunistic aerobic facultative bacteria which become the dominant taxa on just a few days of age^29^, as observed in puppies since D2 and until D21 in this study. Those phenomena result in the reduction of the redox potential of the gut, and of better physicochemical conditions for the establishment and growth of obligate anaerobic bacteria. This neocolonization of the gut explains the increase of bacterial richness over time, together with the enrichment of food sources. In our results, *Moraxellaceae* and *Aerococcaceae,* two aerobic strict families, represented around 25% of the relative abundance of puppies’ microbiota at birth. At D2, they already almost disappeared, while *Enterobacteriaceae* alone, mostly comprised of facultative anaerobic bacteria, such as *E. coli*, represented almost 30% of the relative abundance, inducing the observed decrease in bacterial richness. On later days, the abundance of obligate anaerobes increased, such as *Succinivibrionaceae*, *Bacteroidaceae* and *Bifidobacteriaceae*, while the abundance of facultative aerobes decreased, making our results consistent with the literature in other mammalian species (Fig. 11)^30–32^.

Based on this literature, it is possible to develop a hypothesis of what happened between LBW and normal birth weight (NBW) puppies at birth Despite no significant differences, we observed that LBW puppies had lower abundances of *Aerococcaceae* and *Moraxellaceae* at birth. The lack of those strict aerobic bacteria might have prevented an adequate consumption of oxygen in the GIT during the first days of life. The higher level of oxygen might have induced an earlier and increased colonization of facultative anaerobes, which strived in such an environment compared to the GIT of NBW puppies. The reduced consumption of oxygen also delayed the colonization by strict anaerobes bacteria and subsequently the proper setting of the immune system of newborns^33,34^. Indeed, this abnormal dominance of facultative anaerobic bacteria have been widely reported in preterm infants and is known to induce alteration of the intestinal barrier and immunological functions of the host^33,35,36^. Although, it is important to recall this just an assumption; and it would be interesting to monitor different physico-chemical parameters in an in vitro model or to perform metabolomics analyses (including SCFAs) to have a more accurate picture of the situation.

*Enterobacteriaceae, Clostridiaceae* and *Lachnospiraceae* observed in our study at higher abundance at day 2 and 21 in LBW compared with other groups, are known to be opportunistic bacteria leading to favourable conditions for diseases and specifically inflammatory bowel diseases in the canine species^37–39^. Among those families, we found higher abundances of *E. coli*, *Clostridium perfringens* and *Tyzzerela* in LBW puppies. *E. coli* is demonstrated to be involved in intestinal diseases and systemic infections in newborn puppies^40^, even though this commensal species remains present in high quantities in healthy puppies compared to adult dogs^41^. In many animal species, *Tyzzerela* bacteria are causative agents of Tyzzer’s disease, a usually fatal infectious disease characterized by diarrhea, abdominal distention and hepatic necrotic lesions^42,43^, already described in puppies^44,45^. As *C. perfringens*, this species has also been observed in higher abundances in preterm infants and piglets as well, linked to higher risk of necrotizing enterocolitis^34,46^.

These findings suggest that LBW puppies develop altered gut microbiota during the first days of life, being most probably associated with their higher risk of death. As observed through their significantly lower APGAR score, LBW puppies are weaker than NBW at birth, making them struggle to reach the mammary glands of their mother and consume colostrum^47^. This could explain the initial differences of bacterial composition during the first days of life between LBW and NBW. It remains delicate to determine if the composition of the microbiota induces the weakness of the LBW puppies or the opposite, but those results bring new crucial information on the understanding of the higher risk of mortality of LBW puppies^48^.

Another relevant genus of interest observed in lower abundances in LBW puppies in the present study was *Phascolarctobacterium,* a strict anaerobe which uses succinate to produce acetate and propionate. This genus has been associated with energy metabolism regulation in dogs and humans^49–51^. A recent study highlighted that LBW puppies were at higher risk of getting overweight once adult^52^, thus, the lower abundance of *Phascolarctobacterium* in LBW puppies could be an interesting biomarker to follow. Lower presence of *Fusobacteriaceae* is also of importance, as those bacteria are reported to be related to a “healthy” canine microbiota when compared to the microbiota of dogs suffering from enterocolitis^38^. Those bacteria are anaerobes bacteria with a proteolytic activity and are able to produce SCFAs from protein^53^. Although observed in lower relative abundance, the decrease in *Faecalibacterium*, a butyrate producing bacteria LBW versus NBW is interesting. Indeed, based on these results, it could speculate that there is a modulation in the development of SCFA-producing bacteria, which fermentation products play a crucial role in colonocytes’ metabolism. Also, this genus is highly O2 and pH sensitive, giving credit to the hypothesis of higher level of oxygen in LBW puppies mentioned previously^54^.

Finally, no differences in the microbiota composition between LBW and NBW puppies was observed at D28. All 57 puppies studied remained healthy during the entire studied period. This suggests that the LBW puppies surviving the neonatal period are strong enough to deal with the delayed microbiota development and are able to catch up with other puppies and harbour a “normal” microbiota after a month. As most neonatal deaths occurred during the first weeks of life, with very-low-birth weight ones being at higher risk of death than NBW ones, further studies are needed to explore the development of gut microbiota in dying versus surviving newborn puppies^1,48^.

The results of the present study suggest potential preventive strategies to reduce the mortality of LBW puppies. For example, specific pro and prebiotic strains could be given to LBW puppies as soon as possible to stabilize their microbiota and limit potential dysbiosis. Also, avoiding artificial milk and antibiotic as much as possible is highly recommended, as colostrum is a good source of bacteria for the establishment of the microbiota and antibiotic are known to induce dysbiosis, with in particular the proliferation of facultative anaerobic bacteria^55,56^.

Very few and limited differences between high-birth weight (HBW) and NBW were observed and not recurring from one day to another. The most notable differences were observed at D2 and D21 with higher relative abundances of *Faecalibacterium* and *Bacteroides* respectively. The role of the butyrate-producing *Faecalibacterium* in gut health has been widely studied recently, to the point it became a bioindicator of inflammatory bowel diseases when its abundance decreases, both in humans and dogs^57,58^. Interestingly, this genus seems to be in lower abundance in young puppies compared to adult dogs, so an increase in the abundance of this genus can be linked to the maturity of the puppy^41^. Another genus observed with higher abundance in HBW puppies in this study, *Bacteroides*, is shown to be present in lower abundance in preterm infants^34^. This genus also uses glycans to produce butyrate, reinforces the protection from pathogens in the gut, and as obligate anaerobe, is synonym of the proper maturation of the GIT of the host^59,60^. Thus, the higher abundances of *Faecalibacterium* and *Bacteroides* in HBW puppies during the neonatal period could suggest the maturation of their microbiota happened earlier than in the other puppies. At birth, the HBW puppies might be able to reach the mammary gland with more ease compared to their brethren, allowing them to get more colostrum and potentially, acquiring a more complete microbiota from it^5,61^. At three weeks old, when the first dry diets were presented to puppies, it might be suggested that HBW ingested more food to support the higher needs of their body weight, inducing the increased abundance of *Bacteroides* linked to dry food consumption^23^. Those results would suggest that the early maturation of the GIT microbiota of HBW puppies would give them a better protection against sepsis and other gastrointestinal diseases during the first weeks of life^62^. Since HBW puppies are not specifically studied but usually considered among NBW, it remains difficult to compare those results with literature^63^. If this hypothesis of an early maturation proved to be true, then fecal microbiota transplantation from HBW puppies to LBW puppies could prove to be another effective strategy to stabilize and improve the microbiota stability of LBW puppies using the more mature one of the HBW.

Some limits have to be considered when looking at the results of the present study. First, only 57 puppies were involved, of which 14 were considered LBW and 14 were HBW. While this remains higher than similar studies conducted on other species^34,64^, individual variability of the microbiota composition is a major challenge. On top of it, the LBW studied were in good health and survived the first 2 months of life, meaning their microbiota might not be representative of LBW puppies dying during the neonatal period. All 57 puppies were from the same kennel, which allowed to reduce variability between individuals (same food, same environment), but limit the extrapolation to the whole canine species. Also, previous results highlighted that brethren pups have a closer microbiota composition compared to unrelated ones, meaning the litter effect might have an impact on the results^65^. However, since puppies from a same litter ended up in different quartiles, it allowed to reduce the impact of individual variability of the microbiota composition and confirmed the differences highlighted were most likely a consequence of the birth weight^55,66^.

Nevertheless, while the impact of LBW on puppies’ health had already been studied^3,47,52^, this study is the first one to date to describe differences in the fecal microbial populations based on the birth weight of puppies. Further studies, comparing the microbial profile in LWB puppies dying during the first weeks of life with the surviving ones would be desired to identify bacteria responsible for neonatal mortality in the canine species. Another interesting use of these data would be to develop early life microbial biomarkers per birth weight category to predict the risk of diseases later in life, and to propose therapeutical or nutraceutical strategies to orientate the microbial trajectory.

## Methods

### Ethics approval

The animal study was reviewed and approved by the local ethical committee (Comité d'Éthique en Expérimentation Animale, Science et Santé Animale n°115; reference number: SSA_2020-004, Toulouse, France). All applicable guidelines for the care and use of animals were followed. Written informed consent was obtained from the owner of the kennel for the participation of his animals in this study.

### Animal enrolment and sampling

A total of 57 puppies, born from 11 dams of four different breeds (Australian Shepherd n = 23, Golden Retriever n = 17, Labrador Retriever n = 12 and White Swiss Shepherd n = 5), were recruited within one commercial breeding kennel in France and followed since birth until 2 months of age (Table 3). Among the 11 litters, all puppies suffering from severe health disorder or receiving multiple medical treatments were excluded from the study. Some healthy puppies were also excluded to even the number of puppies per birth weight quartiles. Finally, a mean of 5.2 ± 2.5 (SD) puppies were included within each litter, with a minimum of 2 and a maximum of 10 puppies per litter. Dams gave birth and stayed with their puppies in individual box of a maternity building. For the full duration of gestation and lactation, dogs had wood shaving as bedding material. All dams were fed the same dry puppy diet ad libitum (composition: protein 26%, crude fat 18%, crude fiber 2%, and ash 8%). The same diet mixed with water was presented to puppies between the second and third week of age to allow a transition from milk to solid diet. Then, between 3 and 4 weeks of age (the end of the experiment), puppies had access to the dry diet.

**Table 3:** Characteristics, on the day of mating, of dams included in the study.

Breed	Number of dams	Age of dams (year) (mean)	Dam’s body weight (kg) (mean ± SD)	Dam’s body condition score (BCS on a scale of 9 points^67^) (mean ± SD)	Number of puppies included per litter (mean ± SD)
Australien shepherd	4	3,7 (min = 2.7; max = 4.4)	23.1 ± 2.6	5.8 ± 1.0	5.8 ± 3.1
Golden retriever	3	2,7 (min = 1.2; max = 5.1)	28.1 ± 5.0	5.0 ± 1.5	5.7 ± 2.1
Labrador retriever	2	3,8 (min = 2.7; max = 4.9)	31.2 ± 1.2	7.0 ± 1.4	6.0 ± 2.8
White swiss shepherd	2	3,1 (min = 3.0; max = 3.1)	32.8 ± 2.5	4.0 ± 0.0	2.5 ± 0.7
Total population	11	3,3 ± 1.4	27.7 ± 4.9	5.4 ± 1.4	5.2 ± 2.5

All puppies were born by natural delivery (i.e., no cesarean-section) and remained with their mothers during the entire experiment with the possibility to suckle freely.

### Data recorded

As soon as possible following whelping, APGAR score (vitality index) was assessed for puppies born during daytime according to Veronessi et al.^68^. All puppies were weighed during the first 12 h after birth using a digital scale (EB3 Series, Ohaus, Parsippany, NJ, USA, maximum capacity of 5 kg, precision ± 0.1 g) and birth weights were recorded. Quartiles of birth weight for the four targeted breeds were determined based on a total of 2719 birth weights registered by the breeder during the last 7 years (Table 4). Quartile 1 (Q1) included the 25% puppies with the lightest birth weights, while quartile 4 (Q4) included the 25% heaviest. The thresholds obtained were used to categorize each of the 57 puppies into one of the four quartile groups, based on their breed and birth weight. Puppies in the first quartile were considered LBW puppies, while those in the fourth one were considered HBW. Puppies in the second and third quartiles were fused in a single group, called Q2_3 representing NBW.

**Table 4:** Thresholds of birth weight used to create the four quartiles for each breed, and number of birth weight used to calculate the thresholds.

Birth weight category	LBW	NBW		HBW	Number of birth weights used to calculate thresholds
Breed	Q1 (g)	Q2 (g)	Q3 (g)	Q4 (g)	
Australian shepherd	< 325	325–378	378–426	> 426	663
White swiss shepherd	< 389	389–438	438–498	> 498	249
Golden retriever	< 336	336–382	382–425	> 425	1094
Labrador retriever	< 364	364–410	410–455	> 455	713

Rectal swabs were performed for all puppies (281 total samples) at birth (D0), 2 (D2), 7 (D7), 21 (D21) and 28 (D28) days after birth. Swabs were then stored at − 20 °C immediately after collection for a maximum of 1 month and then at − 80 °C for the rest of time and until further processing.

### DNA extraction and 16S rRNA gene amplification and sequencing

Metagenomic DNA was extracted from rectal swabs using Quick-DNA Fecal/Soil Microbe Miniprep Kit (Zymo Research, Irvine, CA, USA) following the manufacturer’s instructions. Quantification of extracted DNA was checked using a fluorometric method with Quant-iT™ PicoGreen® dsDNA assay kit (Life Technologies, Carlsbad, CA, USA) measured via QuantStudio™3 Real Time PCR System (Thermo Fisher Scientific Inc., Waltham, MA, USA). The V3–V4 region of the 16S rRNA gene was amplified by PCR using universal primers 341F (CCTACGGGAGGCAGCAG)^69^ and 806R (GGACTACNVGGGTWTCTAAT)^70^. PCR amplicons were purified with HighPrep PCR system (Magbio Genomics, Gaithersburg, MD, USA) and used for library construction with the Illumina NEXTflex PCR-Free DNA sequencing kit (Bioo Scientific corp., Austin, TX, USA). Amplicon libraries were sequenced on an Illumina MiSeq 2500 platform (Illumina, San Diego, CA, USA) at GeT-PlaGe INRAE Platform (Toulouse, France) for paired-end fragment sizes of 250 bp. All reagents used were molecular grade. Four samples gave abnormal results and were removed from the study: one at D0, two at D7 and one at D21.

### Analysis of sequencing data

A total of 505 raw fastq files were imported, demultiplexed, quality filtered and dereplicated through high resolution sample inference with DADA2^71^, in QIIME 2 (version 2020.2)^72^. This allowed the identification of amplicon sequence variants (ASVs), under default parameters excluding primers length. ASVs de novo alignment and phylogeny were done respectively with MAFFT and FastTree2^73,74^. Rarefaction curves were checked for full community sampling depth. Taxonomy was assigned to the resulting 16S rRNA marker genes against Greengenes (gg-13-8-99-nb-classifier)/SILVA (v138) using sklearn classifier method according to Bokulich et al.^75^. Raw data were rarefied by removing ASVs present in less than 5 samples.

### Data analysis

All statistical analyses were conducted in R version 4.1.0 (R Core Team, 2021), with resulting *p*-values below 0.05 considered as statistically significant and box-plot and histogram figures drawn with RStudio (version 2022.02.3 Build 492).

ANOVA tests were carried out to evaluate differences in APGAR scores among quartile groups, coupled to a Tukey's post-hoc multiple comparison test to elucidate individual differences between groups when needed. Alpha diversity with Observed ASVs, Shannon and InvSimpson^76^ indices was calculated using the Phyloseq package v1.38.0^77^, and ANOVA coupled to Tukey’s tests were used to evaluate effect of the quartile group on the species richness. Multidimentional scaling (MDS) plots using the phyloseq package were performed to visualize beta diversities between the four quartile groups. To compare the difference in the gut microbiota structure between birth weight quartiles at different time-points, permutational multivariate analysis of variance (PERMANOVA) with 9999 permutations was performed based on Bray–Curtis distances with the Adonis function available in the “vegan” package v2.6–2 of R software. In order to study the differences of bacterial compositions among quartiles, low-abundances reads were first filtered so that ASV present in less than 5 samples were removed. This allowed to carry out sparse Partial Least Squared Discriminant Analysis (sPLSDA) for each bacterial rank at each date using the MixOmics package version 6.22.0^78^. The number of components to keep for the sPLSDA was determined by carrying out predicting receiver operating characteristic (ROC) curves and observing the minimal number of components needed to obtain area under curve (AUC) values higher than 8.0. Variables important to projection (VIPs) were identified using the MixOmics package in R^78^ to better understand the contribution of the said variable in the bacterial composition variation among quartiles. In parallel, a linear mixed effect model with birth weight quartiles, breed, sex and size of litter (defined as the number of puppies born alive in the litter, whether included or not in the study) as fixed effects and the mother of the puppies as random effect, was performed to highlight ASV with significant differences between quartiles at each date, using the lme4 package version 1.1–31. Prior to the analysis, the Geometric Bayesian multiplicative (GBM) method was used to transform ASV with a raw abundance of zero into a value based on intra and inter obeservations between samples. All assumptions of linear regressions; linearity and homoscedasticity (scatter plots), independence of the puppies, normality (central limit theorem^79^) and lack of collinearity between covariates, were validated prior to the analysis. Newly obtained raw abundances data were then transformed using centered log-ratio transformed to better fit the model, using the “compositions” package^80^. Analyses were focused on genus and family ranks. In the end, ASVs with both significant differences between quartiles after the linear mixed effect model analysis and a VIP score above 1.2 were considered to describe differences in the bacterial composition^81^.

## Conclusion

The present study is the first to present differences in the fecal microbiota based on the birth weight of puppies. The main findings report a higher abundance in opportunistic bacteria such as *E. coli*, *Klebsiella* and *Tyzzerella* in LBW puppies, mainly at D2 and D21 after birth as well as a lower bacterial richness at D2. After the third week, no differences were evidenced in the microbiota composition between LBW and NBW puppies, suggesting LBW puppies managed to catch up with their littermates. One possible explanation of this initial difference might be the lack of colostrum intake and physical interactions in LBW puppies with their mother, leading to a weaker bacterial transfer. On the opposite, HBW puppies seem to exhibit an earlier maturation of their microbiota, as shown with higher abundances of *Bacteroides* and *Faecalibacterium* on the first weeks of life. Future studies should be considered to further understand the role of the microbiota composition on newborn dogs’ health, such as its impact on metabolic pathways or how the composition differ when comparing healthy puppies to ill ones. Studying the colostrum and maternal microbiotas may also prove to be useful to further understand the setting of the newborn microbiota.

### Supplementary Information

Supplementary Information.

Supplementary Figure S1.

Supplementary Table S1.

## Data Availability

All data generated or analysed during this study are included in this published article [and its supplementary information files].

## Abbreviations

ASV: Amplicon sequence variant
GIT: Gastrointestinal tract
HBW: High birth weight
LBW: Low birth weight
NBW: Normal birth weight
VIP: Variable importance in projection

## References

[1] Chastant-Maillard S (2017). Reproductive performance and pre-weaning mortality: Preliminary analysis of 27,221 purebred female dogs and 204,537 puppies in France. Reprod. Domest. Anim..

[2] Indrebø A, Trangerud C, Moe L (2007). Canine neonatal mortality in four large breeds. Acta Vet. Scand..

[3] Mugnier A (2019). Birth weight as a risk factor for neonatal mortality: Breed-specific approach to identify at-risk puppies. Prev. Vet. Med..

[4] Groppetti D, Ravasio G, Bronzo V, Pecile A (2015). The role of birth weight on litter size and mortality within 24h of life in purebred dogs: What aspects are involved?. Anim. Reprod. Sci..

[5] Mila H (2015). Immunoglobulin G concentration in canine colostrum: Evaluation and variability. J. Reprod. Immunol..

[6] Sender R, Fuchs S, Milo R (2016). Revised estimates for the number of human and bacteria cells in the body. PLoS Biol..

[7] Suchodolski JS (2011). Intestinal microbiota of dogs and cats: A bigger world than we thought. Vet. Clin. N. Am. Small Anim. Pract..

[8] Henderickx JGE (2021). Maturation of the preterm gastrointestinal tract can be defined by host and microbial markers for digestion and barrier defense. Sci. Rep..

[9] Pammi M (2017). Intestinal dysbiosis in preterm infants preceding necrotizing enterocolitis: A systematic review and meta-analysis. Microbiome.

[10] Cox LM (2014). Altering the intestinal microbiota during a critical developmental window has lasting metabolic consequences. Cell.

[11] Kostic AD (2015). The dynamics of the human infant gut microbiome in development and in progression toward type 1 diabetes. Cell Host Microbe.

[12] Unger S, Stintzi A, Shah P, Mack D, O’Connor DL (2015). Gut microbiota of the very-low-birth-weight infant. Pediatr. Res..

[13] Li N (2019). Characterization of the early life microbiota development and predominant Lactobacillus species at distinct gut segments of low- and normal-birth-weight piglets. Front. Microbiol..

[14] Huang S-M (2020). Perturbation of the lipid metabolism and intestinal inflammation in growing pigs with low birth weight is associated with the alterations of gut microbiota. Sci. Total Environ..

[15] Cai C (2019). Feeding practice influences gut microbiome composition in very low birth weight preterm infants and the association with oxidative stress: A prospective cohort study. Free Radic. Biol. Med..

[16] Guard BC (2017). Characterization of the fecal microbiome during neonatal and early pediatric development in puppies. PLoS ONE.

[17] Del Carro A (2022). The evolution of dam-litter microbial flora from birth to 60 days of age. BMC Vet. Res..

[18] Masuoka H (2017). Transition of the intestinal microbiota of dogs with age. Biosci. Microbiota Food Health.

[19] Saladrigas-García M (2022). An insight into the commercial piglet’s microbial gut colonization: From birth towards weaning. Anim. Microbiome.

[20] Hill CJ (2017). Evolution of gut microbiota composition from birth to 24 weeks in the INFANTMET Cohort. Microbiome.

[21] Frese SA, Parker K, Calvert CC, Mills DA (2015). Diet shapes the gut microbiome of pigs during nursing and weaning. Microbiome.

[22] Fallani M (2011). Determinants of the human infant intestinal microbiota after the introduction of first complementary foods in infant samples from five European centres. Microbiol. Read. Engl..

[23] Li Q, Lauber CL, Czarnecki-Maulden G, Pan Y, Hannah SS (2017). Effects of the dietary protein and carbohydrate ratio on gut microbiomes in dogs of different body conditions. MBio.

[24] Alessandri G (2019). Metagenomic dissection of the canine gut microbiota: Insights into taxonomic, metabolic and nutritional features. Environ. Microbiol..

[25] Vital M, Howe AC, Tiedje JM (2014). Revealing the bacterial butyrate synthesis pathways by analyzing (meta)genomic data. Bio.

[26] Lapébie P, Lombard V, Drula E, Terrapon N, Henrissat B (2019). Bacteroidetes use thousands of enzyme combinations to break down glycans. Nat. Commun..

[27] Litvak Y, Byndloss MX, Bäumler AJ (2018). Colonocyte metabolism shapes the gut microbiota. Science.

[28] Sanidad KZ, Zeng MY (2020). Neonatal gut microbiome and immunity. Curr. Opin. Microbiol..

[29] Buddington RK (2003). Postnatal changes in bacterial populations in the gastrointestinal tract of dogs. Am. J. Vet. Res..

[30] Chaucheyras-Durand F, Sacy A, Karges K, Apper E (2022). Gastro-intestinal microbiota in equines and its role in health and disease: The black box opens. Microorganisms.

[31] Rinninella E (2019). What is the healthy gut microbiota composition? A changing ecosystem across age, environment, diet, and diseases. Microorganisms.

[32] Slifierz MJ, Friendship RM, Weese JS (2015). Longitudinal study of the early-life fecal and nasal microbiotas of the domestic pig. BMC Microbiol..

[33] Arboleya, S., Solís, G., Fernández, N., de los Reyes-Gavilán, C. G. & Gueimonde, M. (2012) Facultative to strict anaerobes ratio in the preterm infant microbiota. *Gut Microbes* **3**, 583–588. 10.4161/gmic.21942 PMC3495798 22922559

[34] Arboleya S (2012). Establishment and development of intestinal microbiota in preterm neonates. FEMS Microbiol. Ecol..

[35] Matamoros S, Gras-Leguen C, Le Vacon F, Potel G, de La Cochetiere M-F (2013). Development of intestinal microbiota in infants and its impact on health. Trends Microbiol..

[36] Magne F (2006). Low species diversity and high interindividual variability in faeces of preterm infants as revealed by sequences of 16S rRNA genes and PCR-temporal temperature gradient gel electrophoresis profiles. FEMS Microbiol. Ecol..

[37] Minamoto Y (2015). Alteration of the fecal microbiota and serum metabolite profiles in dogs with idiopathic inflammatory bowel disease. Gut Microbes.

[38] Suchodolski JS, Dowd SE, Wilke V, Steiner JM, Jergens AE (2012). 16S rRNA gene pyrosequencing reveals bacterial dysbiosis in the duodenum of dogs with idiopathic inflammatory bowel disease. PLoS ONE.

[39] Berry ASF (2019). Gut microbiota features associated with Clostridioides difficile colonization in puppies. PLoS ONE.

[40] Münnich A, Lübke-Becker A (2004). Escherichia coli infections in newborn puppies—Clinical and epidemiological investigations. Theriogenology.

[41] Blake AB (2020). Developmental stages in microbiota, bile acids, and clostridial species in healthy puppies. J. Vet. Intern. Med..

[42] García JA, Navarro MA, Fresneda K, Uzal FA (2022). Clostridium piliforme infection (Tyzzer disease) in horses: Retrospective study of 25 cases and literature review. J. Vet. Diagn. Investig..

[43] Pritt S, Henderson KS, Shek WR (2010). Evaluation of available diagnostic methods for Clostridium piliforme in laboratory rabbits (Oryctolagus cuniculus). Lab. Anim..

[44] Headley SA, Shirota K, Baba T, Ikeda T, Sukura A (2009). Diagnostic exercise: Tyzzer’s disease, distemper, and coccidiosis in a pup. Vet. Pathol..

[45] Young JK, Baker DC, Burney DP (1995). Naturally ocurring Tyzzer’s disease in a puppy. Vet. Pathol..

[46] Cilieborg MS, Boye M, Mølbak L, Thymann T, Sangild PT (2011). Preterm birth and necrotizing enterocolitis alter gut colonization in pigs. Pediatr. Res..

[47] Mila H, Grellet A, Feugier A, Chastant-Maillard S (2015). Differential impact of birth weight and early growth on neonatal mortality in puppies1,2. J. Anim. Sci..

[48] Mugnier A (2020). Low and very low birth weight in puppies: Definitions, risk factors and survival in a large-scale population. BMC Vet. Res..

[49] Pedrogo DAM (2018). Gut microbial carbohydrate metabolism hinders weight loss in overweight adults undergoing lifestyle intervention with a volumetric diet. Mayo Clin. Proc..

[50] Rampelli S (2018). Pre-obese children’s dysbiotic gut microbiome and unhealthy diets may predict the development of obesity. Commun. Biol..

[51] Apper E (2020). Relationships between gut microbiota, metabolome, body weight, and glucose homeostasis of obese dogs fed with diets differing in prebiotic and protein content. Microorganisms.

[52] Mugnier A (2020). Association between birth weight and risk of overweight at adulthood in Labrador dogs. PLoS ONE.

[53] Gethings-Behncke C (2020). Fusobacterium nucleatum in the colorectum and its association with cancer risk and survival: A systematic review and meta-analysis. Cancer Epidemiol. Biomark. Prev..

[54] Khan MT, van Dijl JM, Harmsen HJM (2014). Antioxidants keep the potentially probiotic but highly oxygen-sensitive human gut bacterium *Faecalibacterium* prausnitzii alive at ambient air. PLoS ONE.

[55] Pilla R (2020). Effects of metronidazole on the fecal microbiome and metabolome in healthy dogs. J. Vet. Intern. Med..

[56] Chaitman J (2020). Fecal microbial and metabolic profiles in dogs with acute diarrhea receiving either fecal microbiota transplantation or oral metronidazole. Front. Vet. Sci..

[57] Ferreira-Halder CV, de Faria AVS, Andrade SS (2017). Action and function of *Faecalibacterium* prausnitzii in health and disease. Best Pract. Res. Clin. Gastroenterol..

[58] AlShawaqfeh MK (2017). A dysbiosis index to assess microbial changes in fecal samples of dogs with chronic inflammatory enteropathy. FEMS Microbiol. Ecol..

[59] Beller L (2021). Successional stages in infant gut microbiota maturation. MBio.

[60] Tailford LE, Crost EH, Kavanaugh D, Juge N (2015). Mucin glycan foraging in the human gut microbiome. Front. Genet..

[61] Chastant, S. *et al.* Suckling behavior of puppies during the first 24 hours of life. In *Proceedings of the 22nd Congress of European Veterinary Society for Small Animal Reproduction (EVSSAR)* vol. 54 Suppl. 2 p. 54 (2019).

[62] Tibbs TN, Lopez LR, Arthur JC (2019). The influence of the microbiota on immune development, chronic inflammation, and cancer in the context of aging. Microb. Cell.

[63] Fouhse JM (2020). Outcomes of a low birth weight phenotype on piglet gut microbial composition and intestinal transcriptomic profile. Can. J. Anim. Sci..

[64] Gaukroger CH (2020). Changes in faecal microbiota profiles associated with performance and birthweight of piglets. Front. Microbiol..

[65] Vilson Å (2018). Disentangling factors that shape the gut microbiota in German Shepherd dogs. PLoS ONE.

[66] Bhang, E., Rao, A. & Robinson, A. A potential age-dependent effect of antibiotics on the gut microbiome in dogs with inflammatory bowel disease. *Undergrad. J. Exp. Microbiol. Immunol.* **7**, (2021).

[67] Laflamme D (1997). Development and validation of a body condition score system for dogs. Canine Pract..

[68] Veronesi MC, Panzani S, Faustini M, Rota A (2009). An Apgar scoring system for routine assessment of newborn puppy viability and short-term survival prognosis. Theriogenology.

[69] Muyzer G, de Waal EC, Uitterlinden AG (1993). Profiling of complex microbial populations by denaturing gradient gel electrophoresis analysis of polymerase chain reaction-amplified genes coding for 16S rRNA. Appl. Environ. Microbiol..

[70] Caporaso JG (2011). Global patterns of 16S rRNA diversity at a depth of millions of sequences per sample. Proc. Natl. Acad. Sci. USA.

[71] Callahan BJ (2016). DADA2: High-resolution sample inference from Illumina amplicon data. Nat. Methods.

[72] Bolyen E (2019). Reproducible, interactive, scalable and extensible microbiome data science using QIIME 2. Nat. Biotechnol..

[73] Katoh K, Misawa K, Kuma K, Miyata T (2002). MAFFT: A novel method for rapid multiple sequence alignment based on fast Fourier transform. Nucleic Acids Res..

[74] Price MN, Dehal PS, Arkin AP (2010). FastTree 2–approximately maximum-likelihood trees for large alignments. PLoS ONE.

[75] Bokulich NA (2018). q2-longitudinal: Longitudinal and paired-sample analyses of microbiome data. Systems.

[76] Shannon CE, Weaver W (1949). A mathematical theory of communication. Univ. Ill. Press Urbana.

[77] McMurdie PJ, Holmes S (2013). phyloseq: An R package for reproducible interactive analysis and graphics of microbiome census data. PLoS ONE.

[78] Rohart F, Gautier B, Singh A, Cao K-AL (2017). mixOmics: An R package for ‘omics feature selection and multiple data integration. PLOS Comput. Biol..

[79] Ghasemi A, Zahediasl S (2012). Normality tests for statistical analysis: A guide for non-statisticians. Int. J. Endocrinol. Metab..

[80] van den Boogaart KG, Tolosana-Delgado R (2008). “Compositions”: A unified R package to analyze compositional data. Comput. Geosci..

[81] Akarachantachote N, Chadcham S, Saithanu K (2014). Cutoff threshold of variable importance in projection for variable selection. Int. J. Pure Appl. Math..

