# Supplementary figures and images for: Composition and evolution of the gut microbiota of growing puppies is impacted by their birth weight
Source: Sci Rep. 2023 Sep 7;13:14717. doi: 10.1038/s41598-023-41422-9 (PMC10484951; doi:10.1038/s41598-023-41422-9)

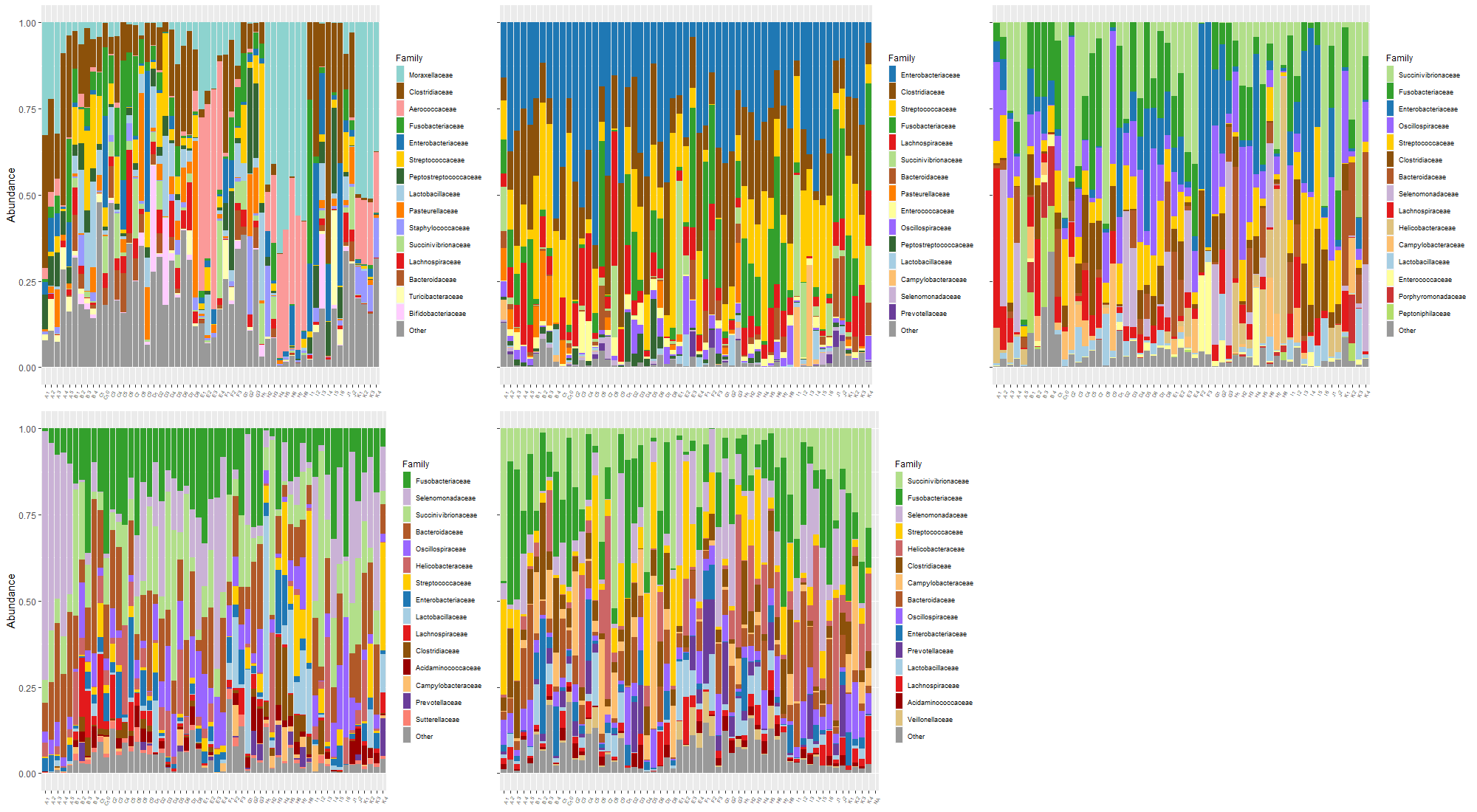

Supplement: Supplementary file 2 — Supplementary Figure S1. [file 41598_2023_41422_MOESM2_ESM.tiff]
